# Simultaneously Transmitting and Reflecting Reconfigurable Intelligent Surfaces Empowered Cooperative Rate Splitting with User Relaying †

**DOI:** 10.3390/e26121019

**Published:** 2024-11-26

**Authors:** Kangchun Zhao, Yijie Mao, Yuanming Shi

**Affiliations:** School of Information Science and Technology, Shanghai Tech University, Shanghai 201210, China; zhaokch12022@shanghaitech.edu.cn (K.Z.); shiym@shanghaitech.edu.cn (Y.S.)

**Keywords:** cooperative rate splitting (CRS), simultaneously transmitting reconfigurable intelligent surface (STAR RIS), rate splitting multiple access (RSMA), max-min fairness (MMF)

## Abstract

In this work, we unveil the advantages of synergizing cooperative rate splitting (CRS) with user relaying and simultaneously transmitting and reflecting reconfigurable intelligent surface (STAR RIS). Specifically, we propose a novel STAR RIS-assisted CRS transmission framework, featuring six unique transmission modes that leverage various combinations of the relaying protocols (including full duplex-FD and half duplex-HD) and the STAR RIS configuration protocols (including energy splitting-ES, mode switching-MS, and time splitting-TS). With the objective of maximizing the minimum user rate, we then propose a unified successive convex approximation (SCA)-based alternative optimization (AO) algorithm to jointly optimize the transmit active beamforming, common rate allocation, STAR RIS passive beamforming, as well as time allocation (for HD or TS protocols) subject to the transmit power constraint at the base station (BS) and the law of energy conservation at the STAR RIS. To alleviate the computational burden, we further propose a low-complexity algorithm that incorporates a closed-form passive beamforming design. Numerical results show that our proposed framework significantly enhances user fairness compared with conventional CRS schemes without STAR RIS or other STAR RIS-empowered multiple access schemes. Moreover, the proposed low-complexity algorithm dramatically reduces the computational complexity while achieving very close performance to the AO method.

## 1. Introduction

In sixth-generation (6G) and beyond mobile communications, an increasing number of devices connect to the wireless network, causing the following problems: (1) Multi-device connections introduce significant multi-user interference, severely degrading system performance. (2) Ensuring a favorable communication environment for each user becomes highly challenging due to the vast number of devices accessing the transmission network. To deal with these problems, a novel non-orthogonal interference management strategy in the physical (PHY) layer named rate-splitting multiple access (RSMA) comes into our sight. It emerges as a promising technique for enhancing spectral efficiency and user fairness [1]. RSMA follows the design principle where user messages are divided into common and private parts at the transmitter. The common parts are encoded into common streams and decoded by multiple users. Meanwhile, the private parts are independently encoded into private streams and decoded only by the corresponding users, following the decoding of the common streams and their subsequent removal through successive interference cancellation (SIC). This empowers RSMA to partially treat interference as noise and partially decode interference. It encompasses various multiple access (MA) schemes like space division MA (SDMA), which fully treats interference as noise, and non-orthogonal MA (NOMA), which fully decodes interference, treating them as special cases [2].

However, as the common streams of RSMA are required to be decoded by multiple users, the achievable rate will be limited by the user with the weakest channel strength. To overcome this limitation, a novel RSMA scheme named cooperative rate splitting (CRS) is proposed in [3,4]. This scheme empowers users with stronger channel conditions, acting as relaying users, to transmit the decoded common stream to users with weaker channel conditions. CRS thereby boosts the received signal strengths at the weaker users and improves the achievable common rate. It not only enhances spectral efficiency but also extends radio coverage, promotes user fairness, and reduces energy consumption [4,5]. There are two transmission phases in CRS, one is the direct transmission phase in which the base station (BS) transmits signals to all users, and the other is the cooperative transmission phase in which the relaying users transmit the decoded common streams to the destination users. According to whether these two phases are executed at the same time, there are two protocols in CRS: *half duplex (HD)* [4] and *full duplex (FD)* [6]. In the *HD* protocol, the direct and cooperative transmission phases are executed in different time slots, while in the *FD* protocol, these two transmission phases are executed at the same time. Both protocols demonstrate significant potential in realizing the aforementioned advantages of CRS.

In parallel, reconfigurable intelligent surface (RIS), gaining increasing attention recently, has been viewed as a promising technique in future wireless networks [7,8,9]. An RIS is a two-dimensional (2D) meta-surface containing numerous passive and low-cost reflecting elements that enable tuning the phase shift of the incident signal. By employing the RIS, the line-of-sight (LoS) channel between the BS and users can be reconfigured, effectively improving the spectral efficiency, and energy efficiency and extending the communication coverage. Nevertheless, conventional RIS can solely reflect signals, limiting its coverage to a $180^{\circ }$ area. This has motivated the emergence of a novel RIS that enables reflection and transmits the incident signal at the same time, which is called simultaneously transmitting and reflecting RIS (STAR RIS) [10] or intelligent omni-surface (IOS) [11]. According to different operating methods, STAR RIS has three protocols: *energy splitting (ES)*, *mode switching (MS)*, and *time switching (TS)*. In the *ES* protocol, all elements can reflect and transmit the incident signal at the same time. In the *MS* protocol, each element either reflects or transmits the incident signal. In the *TS* protocol, the transmission time is split into two time slots. One is the reflection time slot, where all elements reflect the incident signal, and the other is the transmission time slot, where all elements transmit the incident signal. By adjusting the amplitude and phase shift of the reflected and transmitted signals, all three protocols of STAR RIS offer full spatial coverage and additional degrees of freedom (DoF) for the system.

### 1.1. Related Works

In view of the advantages of STAR RIS, many existing works have explored its integration with various MA schemes [12,13,14,15,16,17]. In [12], the authors investigated the coverage range of a two-user STAR RIS-aided orthogonal MA (OMA) or NOMA system. In [13], the beamforming optimization was studied in an uplink STAR RIS-aided NOMA system for outage probability minimization and secrecy capacity maximization. In [14], the sum rate maximization was investigated in a downlink STAR RIS-aided NOMA system, wherein the decoding order and transmit beamforming are jointly optimized. These studies have demonstrated that STAR RIS offers benefits such as extending communication coverage and enhancing the sum rate. Besides, the integration of STAR RIS and RSMA has recently been investigated in some existing works [15,16,17]. In [15], the primary focus was on maximizing the sum secrecy rate in a STAR RIS-aided simultaneous wireless information and power transfer (SWIPT) system using RSMA. In [16], the sum-rate maximization problem for a STAR RIS-aided uplink RSMA system is studied. In [17], efforts were directed toward minimizing the outage probability while considering the spatial correlation among the STAR RIS channels. The aforementioned STAR RIS-aided RSMA works [15,16,17] emphasize the advantages of STAR RIS in enabling full-space wireless networks and enhancing interference management, leading to improvements in both spectral and energy efficiency. However, so far, the integration of STAR RIS and CRS has not been investigated yet. And the performance of max-min fairness for STAR RIS-aided RSMA remains unexplored.

### 1.2. Contributions

Considering the aforementioned advantages of both CRS and STAR RIS, along with the identified research gaps in the current literature, there is a compelling incentive to integrate these two techniques. On one hand, the CRS transmission scheme provides robust and flexible interference management to enhance the performance of STAR RIS. On the other hand, STAR RIS reconfigures and improves wireless channels, thereby further enhancing the performance of CRS. Their integration can lead to a mutually beneficial solution. In this work, we delve into the STAR RIS-aided CRS system, and the main contributions of this paper are summarized as follows:

- We propose a novel downlink STAR RIS-aided CRS transmission framework, empowering a STAR RIS to assist both the direct and cooperative transmission phases of CRS. Within this framework, we investigate six different transmission modes including various combinations of CRS relaying protocols (*HD* and *FD*) and STAR RIS operating protocols (*ES*, *MS*, and *TS*).
- We formulate a new resource allocation problem with the objective of maximizing the minimum user rate. To solve this problem, the STAR RIS passive beamforming, the BS active beamforming, common rate allocation, and time slot allocation (in *HD* or *TS* protocols) are jointly optimized under the transmit power constraint at the BS and the energy conservation constraints at the STAR RIS. Due to the non-convexity of the formulated problem, we propose an alternative optimization (AO) algorithm to solve the problem. This approach involves decomposing the original problem into two subproblems: the STAR RIS passive beamforming optimization and transmit active beamforming optimization. Each subproblem is then solved using a successive convex approximation (SCA)-based method. Through iterative solving of the two subproblems, we attain a near-optimal solution until convergence.
- We further propose a low-complexity algorithm to solve the formulated problem. For the passive beamforming design, we derive a closed-form solution for the STAR RIS passive beamforming based on the gradient descent approach. To ensure the derived solution meets the STAR RIS constraints, we further use the symmetric unitary projection based on singular value decomposition (SVD) to project the solution into the feasible set of constraints. To transmit active beamforming, we use the zero-forcing (ZF) approach to fix the beamforming direction. Subsequently, we simply optimize the power allocation using SCA. Apart from perfect channel state information at the transmitter (CSIT), we also extend the two proposed algorithms to address the ergodic rates (ERs) optimization problems under imperfect CSIT.
- We evaluate the performance of the proposed STAR RIS-aided CRS framework and show the effectiveness of our proposed algorithms by numerical results. Our analysis reveals that the STAR RIS-aided CRS scheme outperforms other STAR RIS-aided MA schemes. We also offer insights into the preferred regions for the six proposed transmission protocols. Moreover, the results demonstrate that our proposed low-complexity algorithm achieves comparable performance while significantly reducing CPU time compared to the AO algorithm.

### 1.3. Organization

The subsequent sections of the paper are organized as follows. Section 2 delineates the system model and formulates the max-min fairness problem. Section 3 details the AO optimization framework for the problem and the proposed low-complexity algorithm. Section 4 presents numerical results. Section 5 concludes the paper.

## 2. System Model and Problem Formulation

**Figure 1:**
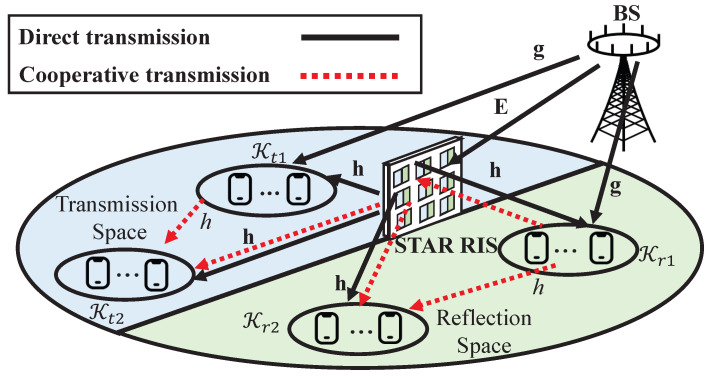
The transmission architecture of the proposed STAR RIS-assisted CRS.

As illustrated in Figure 1, we consider a STAR RIS-assisted multi-user multiple-input single-output (MISO) downlink transmission network, with CRS supported during the transmission. There is a BS equipped with *L* transmit antennas, a STAR RIS containing *N* elements indexed by the set N={1,⋯,N}, and *K* users indexed by the set K={1,2,⋯,K}. Each user is assumed to be low-mobility and equipped with a single transmit antenna and a receive antenna. Given the ability of the STAR RIS to transmit and reflect the incident signal simultaneously, we divide the transmission space into two distinct subspaces, namely, the reflection space and the transmission space. Users in the reflection space receive signals reflected by the STAR RIS and are indexed by the set $K_{r}$. Conversely, users in the transmission space receive signals transmitted by the STAR RIS and are indexed by the set $K_{t}$. The two user sets satisfy $K_{r}\cup K_{t}=K$ and $K_{r}\cap K_{t}=\emptyset$. Both subspaces allow user relaying, which further divides the users in each subspace into two user groups: the relaying user group $K_{i1}$ and the destination user group $K_{i2},i\in \left\{r,t\right\}$. The user groups satisfy $K_{r1}\cap K_{r2}=\emptyset ,K_{t1}\cap K_{t2}=\emptyset$, $K_{r1}\cup K_{r2}=K_{r}$, and $K_{t1}\cup K_{t2}=K_{t}$. For simplicity, we denote the relaying user set and the destination user set in the full space as $K_{1}=K_{t1}\cup K_{r1}$ and $K_{2}=K_{t2}\cup K_{r2}$, respectively. Without loss of generality, we assume users in $K_{1}$ have better channel conditions than users in $K_{2}$.

In this work, we consider a block-fading channel model with *T* transmission blocks indexed by the set T={1,⋯,T}. The channels between the BS and STAR RIS, the BS and user-*k*, STAR RIS and user-*k* are, respectively, denoted by $E\left(t\right)\;\in \;C^{N\times L},\;g_{k}\left(t\right)\;\in \;C^{L\times 1}$, $h_{k}\left(t\right)\;\in \;C^{N\times 1}$, where *t* denotes the *t*-th block, t∈T. Besides, the channel between user-*i* to user-*j* is denoted by $h_{i,j}$. As the pioneer study on STAR RIS-empowered CRS, we employ a simplified CSI model by assuming perfect knowledge of CSI at the BS for all communication links, i.e., perfect CSI at the transmitter (CSIT). The imperfect CSIT setting can be easily extended based on the existing works on RSMA with imperfect CSIT. It is important to highlight that our primary emphasis is on exploring the diverse performance achieved by various transmission modes of STAR RIS-empowered CRS.

### 2.1. Transmission Modes of STAR RIS-Empowered CRS

CRS involves two transmission phases: the direct transmission phase and the cooperative transmission phase. During the direct transmission phase, the BS sends signals to all users. In the cooperative transmission phase, users in $K_{1}$ act as relaying users to transmit the decoded common stream to users in $K_{2}$, utilizing either the *HD* or *FD* protocols. The *FD* protocol allows the simultaneous transmission and reception for relaying users. However, this approach introduces self-interference at the relaying users. In the *HD* protocol, each time block is divided into two consecutive parts, respectively, for the two transmission phases. Let λ(0<λ≤1) denote the fraction of time allocated to the direct transmission phase, and (1−λ) is the fraction allocated to the cooperative transmission phase.

In this paper, both transmission phases of CRS are assisted by the STAR RIS. According to the principle of single-connected STAR RIS in [10], each element of STAR RIS can operate in the reflection mode and/or the transmission mode according to different operating protocols [10].

The reflection and transmission matrices are all diagonal matrices, denoted as $\Theta_{i}=\mathrm{diag}\left(\sqrt{\beta_{1,i}}e^{j\theta_{1,i}},\cdots ,\sqrt{\beta_{N,i}}e^{j\theta_{N,i}}\right)$, i∈{r,t}. In the *ES* protocol, each element of STAR RIS simultaneously reflects and transmits the incident signal. Hence, $\beta_{n,r}$ and $\beta_{n,t}$ should satisfy $\beta_{n,r}+\beta_{n,t}=1$ and $\beta_{n,r},\beta_{n,t}\in \left[0,1\right]$. The *MS* protocol is a special case of *ES* where each element operates in either reflection or transmission mode, resulting in binary values for the amplitude coefficients, i.e., $\beta_{n,r},\beta_{n,t}\in \left\{0,1\right\}$. In the *TS* protocol, each element of STAR RIS switches between the transmission and reflection modes in each transmission block. Define $0\leq \lambda_{r}\leq 1$ as the percentage of time allocated to the reflection mode in each transmission block, and $\left(1-\lambda_{r}\right)$ as the percentage of time allocated to the transmission mode. The *TS* protocol focuses on designing $\lambda_{r}$ and the phase shifts, where $\beta_{n,r}=1,\beta_{n,t}=0$ during the $\lambda_{r}$ percentage of time and $\beta_{n,r}=0,\beta_{n,t}=1$ for the remaining $\left(1-\lambda_{r}\right)$.

**Figure 2:**
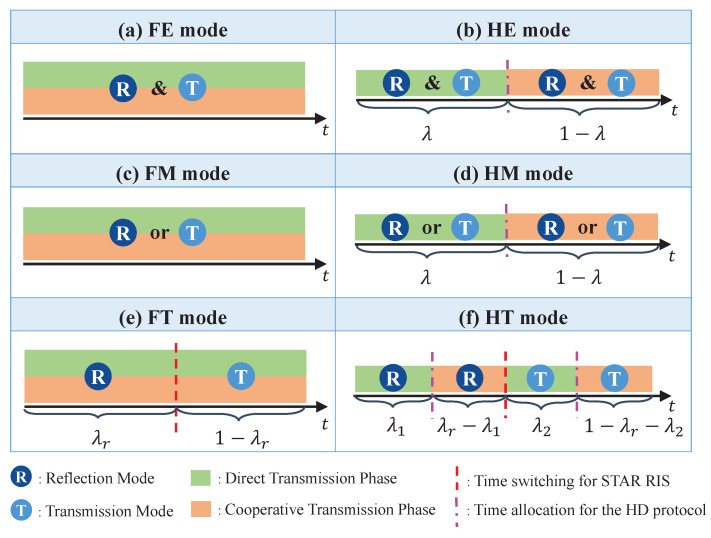
Six transmission modes of the proposed STAR RIS-assisted CRS.

In the proposed STAR RIS-assisted CRS transmission framework, we pair the aforementioned relaying protocols (*HD* and *FD*) with the three STAR RIS operating protocols (*ES*, *MS*, and *TS*), yielding six distinct transmission modes for STAR RIS-assisted CRS. These modes are summarized in Figure 2 and explained as follows:

- *FD-ES (FE)* mode: This refers to the use of the *FD* protocol for CRS and the *ES* protocol for the STAR RIS. As illustrated in Figure 2a, *FE* enables each element of the STAR RIS to operate in both transmission and reflection modes simultaneously in the non-orthogonal direct and cooperative transmission phases.
- *HD-ES (HE)* mode: This refers to the use of the *HD* protocol for CRS and the *ES* protocol for STAR RIS. As illustrated in Figure 2b, *HE* enables each element of the STAR RIS to operate in both transmission and reflection modes simultaneously in the orthogonal direct and cooperative transmission phases.
- *FD-MS (FM)* mode: This refers to the use of the *FD* protocol for CRS and the *MS* protocol for STAR RIS. As illustrated in Figure 2c, *FM* enables each element of the STAR RIS to operate in either transmission or reflection mode in the non-orthogonal direct and cooperative transmission phases.
- *HD-MS (HM)* mode: This refers to the use of the *HD* protocol for CRS and the *MS* protocol for the STAR RIS. As illustrated in Figure 2d, *HM* enables each element of the STAR RIS to operate in either transmission or reflection mode in the orthogonal direct and cooperative transmission phases.
- *FD-TS (FT)* mode: This refers to the use of the *FD* protocol for CRS and the *TS* protocol for the STAR RIS. As illustrated in Figure 2e, each transmission block is split into two time slots based on $\lambda_{r}$ ($0\leq \lambda_{r}\leq 1$). *FT* facilitates all elements of the STAR RIS to function in the reflection mode during the initial portion $\lambda_{r}$ of the time, while they operate in the transmission mode during the subsequent portion $1-\lambda_{r}$ of the time, across the non-orthogonal direct and cooperative transmission phases.
- *HD-TS (HT)* mode: This refers to the use of the *HD* protocol for CRS and the *TS* protocol for the STAR RIS. As illustrated in Figure 2f, each transmission block is split into two time slots based on $\lambda_{r}$: one for the reflection mode and the other for the transmission mode of STAR RIS. Then, each time slot is further split into two orthogonal parts for the direct and cooperative transmission phases of CRS, resulting in a total of four orthogonal transmission slots. Here, we introduce $\lambda_{1}$ ($0<\lambda_{1}\leq \lambda_{r}$) and $\lambda_{2}$ ($0<\lambda_{2}\leq 1-\lambda_{r}$) to represent the time allocation between the two transmission phases in the reflection and transmission modes, respectively.

Hereinafter, we use superscript *j* to represent the selected transmission mode, where j∈{FE,FM,FT,HE,HM,HT}.

### 2.2. Transmit Signal Model

For all six transmission modes described in the previous subsection, the primary differences among them lie in the received signals and the approaches employed to process the received signals. The transmit signals in both the direct and cooperative transmission phases remain consistent, as specified in this subsection.

#### 2.2.1. Direct Transmission Phase

We denote $W_{k}$ as the message intended for user-*k*. At the BS, according to the principle of one-layer RSMA [2,18], $W_{k}$ is split into a common sub-message $W_{c,k}$ and a private sub-message $W_{p,k}$. All common sub-messages $W_{c,1},\cdots ,W_{c,K}$ are collected and then encoded into a common stream $s_{0}$, which is required to be decoded by all users. The individual private sub-message $W_{p,k}$ is independently encoded into a private stream $s_{k}$, which is required to be decoded by user-*k* only. Denote $s={\left[s_{0},s_{1},\ldots ,s_{K}\right]}^{T}$ and $P=\left[p_{0},p_{1},\ldots ,p_{K}\right]\in C^{L\times (K+1)}$, respectively, as the stream vector and the transmit beamforming matrix, where $p_{k}\in C^{L\times 1}$. The transmit signal at the BS in the direct transmission phase is given by

(1) $$x^{\left[1\right]}=Ps=\sum_{k=0}^{K}p_{k}s_{k},$$

where the superscript [1] denotes the direct transmission phase. Here, assuming $E\{ss^{H}\}=I$, the transmit power of the BS is limited by $\mathrm{tr}\left(PP^{H}\right)\leq P_{t}$, where $P_{t}$ is the maximum transmit power at the BS.

#### 2.2.2. Cooperative Transmission Phase

Users in $K_{1}$ employ the decode-and-forward (DF) protocol to decode, re-encode, and subsequently transmit the decoded $s_{0}$ to users in $K_{2}$. The transmit signal at the relaying user-*k* in the cooperative transmission phase is

(2) $$x_{k}^{\left[2\right]}=\sqrt{P_{k}}s_{0},$$

where the superscript [2] denotes the cooperative transmission phase and $P_{k}$ is the relaying transmit power at user-*k*.

### 2.3. Received Signal Model for FD-Based Transmission Modes

In the FD-based transmission modes (*FE*, *FM*, and *FT*), both the direct and cooperative transmission phases share the same radio resources. Self-interference is inevitably among the relaying users in $K_{1}$ as they simultaneously receive signals from the BS and transmit the common stream $s_{0}$ to users in $K_{2}$, [19,20]. We denote the self-interference channel as $I_{k}\left(t\right)$, which follows the same distribution $I_{k}\left(t\right)∼\mathrm{CN}\left(0,\Omega_{I}^{2}\right)$ for all $k\in K_{1}$. The signal received by users in $K_{2}$ from $K_{1}$, denoted as ${\hat{s}}_{0}\left(t\right)$, is a delayed version of $s_{0}$, where ${\hat{s}}_{0}\left(t\right)=s_{0}\left(t-t_{d}\right)$. Here, $t_{d}$ represents the processing time for users in $K_{1}$ to decode and forward $s_{0}$. To ensure that the relaying users transmit and receive ${\hat{s}}_{0}\left(t\right)$ within the same block, we assume that $t_{d}$ is much smaller than one block period [20,21]. In this work, we focus on optimizing P, $\Theta_{r}$ and $\Theta_{t}$ in each transmission block. Hence, we omit *t* in the following. Next, we will specify the received signals and achievable rates for *FE*, *FM*, and *FT*, respectively.

#### 2.3.1. FE/FM

When j=FE or j=FM, the signal received at each relaying user-*k* in $K_{1}$ is given by

(3) $$\begin{array}{c} y_{k}^{j}=\underset{{\tilde{g}}_{k,i}^{H}}{\left(\underset{︸}{g_{k}^{H}+h_{k}^{H}\Theta_{i}E}\right)}x^{\left[1\right]}+I_{k}\sqrt{P_{k}}{\hat{s}}_{0}+n_{k},i\in \left\{r,t\right\}, \end{array}$$

where $n_{k}$ is the additive white Gaussian noise (AWGN) which follows $n_{k}∼\mathrm{CN}\left(0,\sigma^{2}\right)$ and $I_{k}{\sqrt{P}}_{k}{\hat{s}}_{0}$ is the self-interference suffered at user-*k*. Each user-*k* in $K_{1}$ sequentially decodes the common stream $s_{0}$ and the private stream $s_{k}$ with the assistance of SIC. Let ${\tilde{g}}_{k,i}$ denote the effective channel between the BS and user-*k*, the SINRs of decoding $s_{0}$ and $s_{k}$ at user-*k* in $K_{1}$ are, respectively, given as

(4) $$\begin{array}{cc} \; & \gamma_{c,k}^{\mathrm{FD}}=\gamma_{c,k}^{\mathrm{FD},[1]}=\frac{{\left|{\tilde{g}}_{k,i}^{H}p_{0}\right|}^{2}}{\sum_{m\in K}{\left|{\tilde{g}}_{k,i}^{H}p_{m}\right|}^{2}+{\left|I_{k}\right|}^{2}P_{k}+\sigma^{2}}, \end{array}$$

(5) $$\begin{array}{cc} \; & \gamma_{k}^{\mathrm{FD}}=\frac{{\left|{\tilde{g}}_{k,i}^{H}p_{k}\right|}^{2}}{\sum_{m\in K,m\neq k}{\left|{\tilde{g}}_{k,i}^{H}p_{m}\right|}^{2}+{\left|I_{k}\right|}^{2}P_{k}+\sigma^{2}}. \end{array}$$

Each user-*k* in $K_{2}$ receives signals from both the BS and the relaying users in $K_{1}$, which are given as

(6) $$\begin{array}{c} y_{k}^{j}={\tilde{g}}_{k,i}^{H}x^{\left[1\right]}+\sum_{m\in K_{1}}\underset{{\tilde{h}}_{m,k,i}}{\left(\underset{︸}{h_{m,k}+h_{k}^{H}\Theta_{i}^{H}h_{m}}\right)}x_{m}^{\left[2\right]}+n_{k},\;i\in \left\{r,t\right\}. \end{array}$$

Each user in $K_{2}$ then employs the maximal ratio combining (MRC) approach to ensure the received signals are properly co-phased and merged [22]. Denote the effective channel between user-*m* and user-*n* as ${\tilde{h}}_{m,n,i}$, the SINRs of decoding the $s_{0}$ and $s_{k}$ at user-*k* in $K_{2}$ are given as

(7) $$\begin{array}{cc} \; & \gamma_{c,k}^{\mathrm{FD}}=\underset{\gamma_{c,k}^{\mathrm{FD},[1]}}{\underset{︸}{\frac{{\left|{\tilde{g}}_{k,i}^{H}p_{0}\right|}^{2}}{\sum_{m\in K}{\left|{\tilde{g}}_{k,i}^{H}p_{m}\right|}^{2}+\sigma^{2}}}}+\underset{\gamma_{c,k}^{\mathrm{FD},[2]}}{\underset{︸}{\frac{\sum_{m\in K_{1}}{\left|{\tilde{h}}_{m,k,i}\right|}^{2}P_{m}}{\sigma^{2}}}}, \end{array}$$

(8) $$\begin{array}{cc} \; & \gamma_{k}^{\mathrm{FD}}=\frac{{\left|{\tilde{g}}_{k,i}^{H}p_{k}\right|}^{2}}{\sum_{m\in K,m\neq k}{\left|{\tilde{g}}_{k,i}^{H}p_{m}\right|}^{2}+\sigma^{2}}. \end{array}$$

With the SINRs of $s_{0}$ and $s_{k}$ in (4), (5), (7) and (8), the achievable common and private rates are obtained as $R_{c,k}^{j}=\log_{2}\left(1+\gamma_{c,k}^{\mathrm{FD}}\right)$ and $R_{k}^{j}=\log_{2}\left(1+\gamma_{k}^{\mathrm{FD}}\right)$, j∈{FE,FM}. To guarantee all users decode the common stream, the following condition should satisfy [2]:

(9) $$R_{c}^{j}=\min\left\{R_{c,k}^{j}|k\in K\right\},$$

$R_{c}^{j}$ is shared by all users, we denote $C_{k}$ as the part of $R_{c}^{j}$ that is allocated to user-*k*, it satisfies $\sum_{k\in K}C_{k}\leq R_{c}^{j}$. Hence, the total achievable rate of user-*k* is

(10) $$R_{tot,k}^{j}=R_{k}^{j}+C_{k},\forall k\in K.$$

Note that Equations (9) and (10) remain applicable for other transmission modes by setting *j* to FT/HE/HM/HT. In the following discussion of these transmission modes, the equations will not be repeated.

#### 2.3.2. FT

In *FT* mode, there are two time slots: all elements of STAR RIS operate in the reflection mode during the first time slot ($\lambda_{r}$) while all elements of STAR RIS operate in the transmission mode during the second time slot ($1-\lambda_{r}$). We denote the SINRs for decoding $s_{0}$ and $s_{k}$ in the reflection and transmission time slots, respectively, as $\gamma_{c,k}^{\mathrm{FD},i}$ and $\gamma_{k}^{\mathrm{FD},i}$, where i∈{r,t}. In the reflection time slot, users in $K_{r}$ receive signals from the BS and the STAR RIS while users in $K_{t}$ receive signals from the BS only. The corresponding SINRs are given as

(11) $$\begin{array}{cc} \gamma_{c,k}^{\mathrm{FD},r} & =\gamma_{c,k}^{\mathrm{FD}}{|}_{\Theta_{i}=\Theta_{r}},\;\;\gamma_{k}^{\mathrm{FD},r}=\gamma_{k}^{\mathrm{FD}}{|}_{\Theta_{i}=\Theta_{r}},k\in K_{r},\\ \gamma_{c,k}^{\mathrm{FD},r} & =\gamma_{c,k}^{\mathrm{FD}}{|}_{\Theta_{i}=0},\;\;\gamma_{k}^{\mathrm{FD},r}=\gamma_{k}^{\mathrm{FD}}{|}_{\Theta_{i}=0},k\in K_{t}, \end{array}$$

where $\gamma_{c,k}^{\mathrm{FD}}$ and $\gamma_{k}^{\mathrm{FD}}$ are defined in (4) and (5), respectively. In the transmission time slot, users in $K_{r}$ receive signals from the BS only while users in $K_{t}$ receive signals from both BS and STAR RIS. The corresponding SINRs are given as

(12) $$\begin{array}{cc} \gamma_{c,k}^{\mathrm{FD},t} & =\gamma_{c,k}^{\mathrm{FD}}{|}_{\Theta_{i}=0},\;\;\gamma_{k}^{\mathrm{FD},t}=\gamma_{k}^{\mathrm{FD}}{|}_{\Theta_{i}=0},k\in K_{r},\\ \gamma_{c,k}^{\mathrm{FD},t} & =\gamma_{c,k}^{\mathrm{FD}}{|}_{\Theta_{i}=\Theta_{t}},\;\;\gamma_{k}^{\mathrm{FD},t}=\gamma_{k}^{\mathrm{FD}}{|}_{\Theta_{i}=\Theta_{t}},k\in K_{t}. \end{array}$$

The achievable common and private rates for user-*k* are given as

(13) $$\begin{array}{cc} R_{c,k}^{\mathrm{FT}} & =\lambda_{r}\log_{2}\left(1+\gamma_{c,k}^{\mathrm{FD},r}\right)+\left(1-\lambda_{r}\right)\log_{2}\left(1+\gamma_{c,k}^{\mathrm{FD},t}\right),\\ R_{k}^{\mathrm{FT}} & =\lambda_{r}\log_{2}\left(1+\gamma_{k}^{\mathrm{FD},r}\right)+\left(1-\lambda_{r}\right)\log_{2}\left(1+\gamma_{k}^{\mathrm{FD},t}\right). \end{array}$$

By substituting $R_{c,k}^{\mathrm{FT}}$ and $R_{k}^{\mathrm{FT}}$ into (9) and (10), we obtain the corresponding achievable rate of each user in the FT mode.

### 2.4. Received Signal Model for HD-Based Transmission Modes

In *HD*-based transmission modes, the direct and cooperative transmission phases are orthogonal in each transmission block.

In contrast to *FD*-based modes, there is no self-interference at relaying users, but the transmission rates for the direct and transmission phases are scaled by the pre-log factors λ(0<λ≤1) and (1−λ), respectively. In the following, we specify the received signals and achievable rates for *HE*, *HM*, and *HT*.

#### 2.4.1. HE/HM

In the direct transmission phase, the signal received at user-*k* is

(14) $$\begin{array}{c} y_{k}^{\mathrm{HD},[1]}={\tilde{g}}_{k,i}^{H}x^{\left[1\right]}+n_{k},\forall k\in K,j\in \left\{\mathrm{HE},\mathrm{HM}\right\}. \end{array}$$

The SINRs of decoding $s_{0}$ and $s_{k}$ in the direct transmission phase are given as

(15) $$\begin{array}{cc} \; & \gamma_{c,k}^{\mathrm{HD},[1]}=\frac{{\left|{\tilde{g}}_{k,i}^{H}p_{0}\right|}^{2}}{\sum_{m\in K}{\left|{\tilde{g}}_{k,i}^{H}p_{m}\right|}^{2}+\sigma^{2}}, \end{array}$$

(16) $$\begin{array}{c} \gamma_{k}^{\mathrm{HD}}=\frac{{\left|{\tilde{g}}_{k,i}^{H}p_{0}\right|}^{2}}{\sum_{m\in K,m\neq k}{\left|{\tilde{g}}_{k,i}^{H}p_{m}\right|}^{2}+\sigma^{2}}. \end{array}$$

In the cooperative transmission phase, users in $K_{1}$ forward the decoded $s_{0}$ to users in $K_{2}$. The signal received by user-*k* in $K_{2}$ in the cooperative transmission phase is

(17) $$\begin{array}{c} y_{k}^{\mathrm{HD},[2]}=\sum_{m\in K_{1}}{\tilde{h}}_{m,k,i}\sqrt{P_{m}}s_{0}+n_{k},\forall k\in K_{2}. \end{array}$$

The SINR of decoding $s_{0}$ at user-*k* in $K_{2}$ in the cooperative transmission phase is given as

(18) $$\begin{array}{c} \gamma_{c,k}^{\mathrm{HD},[2]}=\frac{\sum_{m\in K_{1}}{\left|{\tilde{h}}_{m,k,i}\right|}^{2}P_{m}}{\sigma^{2}}. \end{array}$$

With the SINR in (15), (16) and (18), we obtain the achievable common and private rates in *HE* and *HM* modes (j∈HE/HM) as

(19) $$\begin{array}{cc} R_{c,k}^{j} & =\lambda \log_{2}\left(1+\gamma_{c,k}^{\mathrm{HD},[1]}\right),\forall k\in K_{1}\\ R_{c,k}^{j} & =\lambda \log_{2}\left(1+\gamma_{c,k}^{\mathrm{HD},[1]}\right)+\left(1-\lambda \right)\log_{2}\left(1+\gamma_{c,k}^{\mathrm{HD},[2]}\right),\forall k\in K_{2} \end{array}$$

and

(20) $$R_{k}^{j}=\lambda \log_{2}\left(1+\gamma_{k}^{\mathrm{HD}}\right).$$

By substituting (19) and (20) into (9) and (10), we obtain the corresponding achievable rate of each user in the *HE* and *HM* modes.

#### 2.4.2. HT

In the *HT* mode, there are four time slots: two for direct transmission with STAR RIS reflection or transmission, and two for cooperative transmission with STAR RIS reflection or transmission. We use $\gamma_{c,k}^{\mathrm{HD},[s],i}$ and $\gamma_{k}^{\mathrm{HD},i}$, i∈{r,t}, s∈{1,2} to denote the SINRs of decoding $s_{0}$ and $s_{k}$ in the direct or cooperative transmission phase. The superscript *i* indicates whether it is in the reflection or transmission time slot. Specifically, in the two reflection time slots, respectively, for cooperative and transmission phases, we have

(21) $$\begin{array}{cc} \gamma_{c,k}^{\mathrm{HD},[s],r} & =\gamma_{c,k}^{\mathrm{HD},[s]}{|}_{\Theta_{i}=\Theta_{r}},\;\;\gamma_{k}^{\mathrm{HD},r}=\gamma_{k}^{\mathrm{HD}}{|}_{\Theta_{i}=\Theta_{r}},k\in K_{r},\\ \gamma_{c,k}^{\mathrm{HD},[s],r} & =\gamma_{c,k}^{\mathrm{HD},[s]}{|}_{\Theta_{i}=0},\;\;\gamma_{k}^{\mathrm{HD},r}=\gamma_{k}^{\mathrm{HD}}{|}_{\Theta_{i}=0},k\in K_{t}, \end{array}$$

where $\gamma_{c,k}^{\mathrm{HD},[s]},s\in \left\{1,2\right\}$ and $\gamma_{k}^{\mathrm{HD}}$ are defined in (15), (18) and (16), respectively. In the two transmission time slots, respectively, for cooperative and transmission phases, we have

(22) $$\begin{array}{cc} \gamma_{c,k}^{\mathrm{HD},[s],t} & =\gamma_{c,k}^{\mathrm{HD},[s]}{|}_{\Theta_{i}=0},\;\;\gamma_{k}^{\mathrm{HD},t}=\gamma_{k}^{\mathrm{HD}}{|}_{\Theta_{i}=0},k\in K_{r},\\ \gamma_{c,k}^{\mathrm{HD},[s],t} & =\gamma_{c,k}^{\mathrm{HD},[s]}{|}_{\Theta_{i}=\Theta_{t}},\;\;\gamma_{k}^{\mathrm{HD},t}=\gamma_{k}^{\mathrm{HD}}{|}_{\Theta_{i}=\Theta_{t}},k\in K_{t}. \end{array}$$

Hence, the achievable common and private rates for user-*k* are given as

(23) $$\begin{array}{cc} R_{c,k}^{\mathrm{HT}} & =\lambda_{1}\log_{2}\left(1+\gamma_{c,k}^{\mathrm{HD},[1],r}\right)+\lambda_{2}\log_{2}\left(1+\gamma_{c,k}^{\mathrm{HD},[1],t}\right),\forall k\in K_{1},\\ R_{c,k}^{\mathrm{HT}} & =\lambda_{1}\log_{2}\left(1+\gamma_{c,k}^{\mathrm{HD},[1],r}\right)+\left(\lambda_{r}-\lambda_{1}\right)\log_{2}\left(1+\gamma_{c,k}^{\mathrm{HD},[2],r}\right)\\ \; & +\;\lambda_{2}\log_{2}\left(1+\gamma_{c,k}^{\mathrm{HD},[1],t}\right)+\left(1-\lambda_{r}-\lambda_{2}\right)\log_{2}\left(1+\gamma_{c,k}^{\mathrm{HD},[2],t}\right),\;\forall k\in K_{2}. \end{array}$$

(24) $$R_{k}^{\mathrm{HT}}=\lambda_{1}\log_{2}\left(1+\gamma_{k}^{\mathrm{HD},r}\right)+\lambda_{2}\log_{2}\left(1+\gamma_{k}^{\mathrm{HD},t}\right).$$

### 2.5. Problem Formulation

In this work, we aim to jointly optimize the active beamforming P at the BS, the common rate allocation $c=[C_{1},\cdots ,C_{K}]$, as well as the passive beamforming $\Theta_{r}$, $\Theta_{t}$ at the STAR RIS. For the *HD*-based transmission modes, the corresponding time allocation coefficients are also jointly optimized. Our objective is to maximize the minimum user rate (max-min rate) among all users subject to the transmit power constraint. The formulated problems for the six transmission modes of the proposed STAR RIS-aided CRS model are illustrated as follows:

#### 2.5.1. FE

j=FE, the optimization problem is formulated as

(25a)PFE:maxP,c,Θr,Θtmink∈KRtot,kFE(25b)s.t.∑k∈KCk≤Rcj,Ck≥0,∀k∈K(25c)θn,i∈[0,2π),∀n∈N,i∈{r,t},(25d)trPPH≤Pt(25e)βn,r,βn,t∈[0,1],βn,r+βn,t=1,∀n∈N,

where constraint (25b) guarantees the common stream and is successfully decoded by all users. Constraints (25c) and (25e) are the constraints for the phase shift and amplitude ranges for each element of the STAR RIS, respectively. (25d) limits the transmit power at the BS.

#### 2.5.2. FM

j=FM, the formulated problem is given as

(26a)PFM:maxP,c,Θr,Θtmink∈KRtot,kFM s.t.(25b)–(25d)(26b)βn,r,βn,t∈{0,1},βn,r+βn,t=1,∀n∈N.

#### 2.5.3. FT

j=FT, the time allocation variable $\lambda_{r}$ is jointly optimized with the beamforming matrices and the common rate allocation. The optimization problem is

(27a)PFT:maxP,c,Θr,Θt,λrmink∈KRtot,kFT s.t.(25b)–(25d),(27b)βn,r,βn,t∈[0,1],∀n∈N,(27c)0≤λr≤1.

#### 2.5.4. HE

j=HE, the time allocation variable λ is jointly optimized with the beamforming matrices and the common rate allocation. The formulated problem is

(28a)PHE:maxP,c,Θr,Θt,λmink∈KRtot,kHE s.t.(25b)–(25e)(28b)0<λ≤1.

#### 2.5.5. HM

j=HM, the corresponding optimization problem is formulated as

(29) $$\begin{array}{cc} P_{\mathrm{HM}}: & \max_{P,c,\Theta_{r},\Theta_{t},\lambda }\;\;\min_{k\in K}\;\;R_{tot,k}^{\mathrm{HM}}\\ s.t. & (25b)\text{–}(25d),\;(26b),\;(28b). \end{array}$$

#### 2.5.6. HT

j=HT, $\lambda_{r},\lambda_{1},\lambda_{2}$ are jointly optimized. The corresponding optimization problem is formulated as

(30a)PHT:maxP,c,Θr,Θt,λr,λ1,λ2mink∈KRtot,kHT s.t.(25b)–(25d),(27b),(27c)(30b)0<λ1≤λr,0<λ2≤1−λr.

From problems (25)–(30) for the six transmission modes, we observe that P, $\Theta_{r}$, $\Theta_{t}$ are highly coupled in the non-convex and fractional expressions for the achievable common and private rates. Besides, problem (26) and (29) involve integer programming due to (25b). All problems are highly non-convex and difficult to solve. In the next section, we will propose two algorithms to solve the problems.

## 3. Proposed Optimization Frameworks

To solve the aforementioned six non-convex optimization problems, in this section, we first propose an AO framework that alternatively optimizes the STAR RIS passive beamforming matrices $\Theta_{r}$ and $\Theta_{t}$, and the remaining variables. To ease the computational complexity, we further propose a low-complexity algorithm to address the optimization problems. In the end, we extend the proposed optimization framework to the imperfect CSIT scenario.

### 3.1. Proposed AO Algorithm

We divide the original non-convex problems into two subproblems, one for the STAR RIS passive beamforming design, and the other for the joint BS active beamforming, common rate, and time allocation design. The approaches for solving the two subproblems are detailed in the following:

#### 3.1.1. STAR RIS Passive Beamforming Optimization

With the transmit beamforming P, common rate allocation c and time allocation variables $\lambda_{t}$, $\lambda_{1}$ and $\lambda_{2}$ fixed, we focus on optimizing $\Theta_{r}$ and $\Theta_{t}$. First, we introduce an auxiliary variable $t^{j}$ to represent the objective function for the transmission mode *j*, leading to the following constraint:

(31) $$R_{p,k}^{j}+C_{k}\geq t^{j},\forall k\in K,j\in \left\{\mathrm{FE},\mathrm{FM},\mathrm{FT},\mathrm{HE},\mathrm{HM},\mathrm{HT}\right\}.$$

Although the achievable rate expressions may vary across different transmission modes, our main focus is the SINR expressions as the optimization variables $\Theta_{r}$ and $\Theta_{t}$ are embedded solely within the SINR. Note that the non-convexity of the SINR expressions remains consistent across all six transmission modes. The key distinction in optimizing the STAR RIS across these modes lies in the constraints imposed by the *ES*, *MS*, and *TS* protocols.

In the following, we begin by illustrating the SCA-based method employed to address the non-convexity of the SINR expressions, using the *FE* mode as an example. Following this, we elaborate on the penalty methods proposed to manage the STAR RIS constraints for the *ES*, *MS*, and *TS* protocols, respectively.

- **Step 1.1: The SCA method for non-convex SINRs**:

Let $\psi_{n,i}=\sqrt{\beta_{n,i}}e^{j\theta_{n,i}},n\in N,i\in \left\{r,t\right\}$ denote each element of $\Theta_{r}$ and $\Theta_{t}$. With $\psi_{i}={\left[\psi_{1,i},\ldots ,\psi_{N,i}\right]}^{T}$, we have $g_{k}^{H}p_{m}+h_{k}^{H}\Theta_{i}Ep_{m}={\bar{g}}_{km}+s_{km}^{H}\psi_{i}$, $h_{n}^{H}\Theta_{i}h_{m}={\bar{h}}_{m,n}^{H}\psi_{i}$, where ${\bar{g}}_{km}=g_{k}^{H}p_{m}$, $s_{km}={\left(\mathrm{diag}\left(h_{k}^{H}\right)Ep_{m}\right)}^{*}$ and ${\bar{h}}_{m,n}={\left(\mathrm{diag}\left(h_{n}^{H}\right)h_{m}\right)}^{*}\in C^{N\times 1}$. In this way, the optimization variables are represented by $\psi_{r}$ and $\psi_{t}$.

Taking the *FE* problem (25) as an example, we first introduce slack variables $\delta ={\left[\delta_{1},\ldots ,\delta_{K}\right]}^{T}$ and $\delta_{c}={\left[\delta_{c,1},\ldots ,\delta_{c,K}\right]}^{T}$ to, respectively, denote the SINRs of $s_{k}$ and $s_{0}$ in the direct transmission phase. Furthermore, we introduce $\xi =[\xi_{1},\ldots ,\xi_{K_{2}}]$ to denote the SINRs of the destination users in the cooperative transmission phase, where $K_{2}$ is the number of users in $K_{2}$. The optimization problem (25) for updating the STAR RIS passive beamforming matrices is then equivalently transformed into

(32a)PFE−1:maxtFE,ψr,ψt,δ,δc,ξtFE(32b)s.t.log2(1+δk)+Ck≥tFE,∀k∈K,(32c)log2(1+δc,k)≥∑m∈KCm,k∈K1,(32d)log2(1+δc,k+ξk)≥∑m∈KCm,k∈K2,(32e)δc,k≤γc,kFD,[1],∀k∈K,(32f)δk≤γkFD,∀k∈K,(32g)ξk≤γc,kFD,[2],∀k∈K2, (25e).

For constraints (32e) and (32f), we further introduce slack variables $\eta ={\left[\eta_{1},\ldots ,\eta_{K}\right]}^{T}$ and $\eta_{c}={\left[\eta_{c,1},\ldots ,\eta_{c,K}\right]}^{T}$ to, respectively, denote the denominators of the SINRs for $s_{0}$ and $s_{k}$. (32e) and (32f) are equivalently transformed to

(33a)ηc,k≥de(γc,kFD,[1]),∀k∈K(33b)ηk≥de(γkFD),∀k∈K(33c)δc,kηc,k≤g¯k0+sk0Hψi2,(33d)δkηk≤g¯kk+skkHψi2,

where de(·) is an operator defined to extract the denominator of a fraction, i.e., $\mathrm{de}(\frac{a}{b})=b$. For constraint (33c), the left-hand side (LHS) equals to $\delta_{c,k}\eta_{c,k}=\frac{1}{4}{\left(\delta_{c,k}+\eta_{c,k}\right)}^{2}-\frac{1}{4}{\left(\delta_{c,k}-\eta_{c,k}\right)}^{2}$. By further approximating $\delta_{c,k}\eta_{c,k}$ at the point $\left(\delta_{c,k}^{\left[z\right]},\eta_{c,k}^{\left[z\right]}\right)$ in iteration [z] using the first-order Taylor approximation of ${\left(\delta_{c,k}-\eta_{c,k}\right)}^{2}$, we have

(34) $$\begin{array}{cc} \delta_{c,k}\eta_{c,k}\leq & \frac{1}{4}{\left(\delta_{c,k}+\eta_{c,k}\right)}^{2}-\frac{1}{2}\left(\delta_{c,k}^{\left[z\right]}-\eta_{c,k}^{\left[z\right]}\right)\left(\delta_{c,k}-\eta_{c,k}\right)+\frac{1}{4}{\left(\delta_{c,k}^{\left[z\right]}-\eta_{c,k}^{\left[z\right]}\right)}^{2}≜\nu \left(\delta_{c,k}^{\left[z\right]},\eta_{c,k}^{\left[z\right]}\right). \end{array}$$

We also approximate the right-hand side (RHS) of (33c) at $\psi_{i}^{\left[z\right]}$ by the first-order Taylor approximation of ${\left|{\bar{g}}_{k0}+s_{k0}^{H}\psi_{i}\right|}^{2}$ as

(35) $$\begin{array}{cc} {\left|{\bar{g}}_{k0}+s_{k0}^{H}\psi_{i}\right|}^{2}\geq & 2ℜ\left\{{\left(s_{k0}^{H}\psi_{i}^{\left[z\right]}+{\bar{g}}_{k0}\right)}^{H}s_{k0}^{H}\psi_{i}\right\}-|s_{k0}^{H}\psi_{i}{|}^{2}+{\left|{\bar{g}}_{k0}\right|}^{2}≜\varpi \left(\psi_{i}^{\left[z\right]},\psi_{i},{\bar{g}}_{k0},s_{k0}\right). \end{array}$$

Based on this approach, constraints (33c) and (33d) are transformed to

(36a)ν(δc,k[z],ηc,k[z])≤ϖ(ψi[z],ψi,g¯k0,sk0),∀k∈K1,(36b)ν(δk[z],ηk[z])≤ϖ(ψi[z],ψi,g¯kk,skk),∀k∈K.

And constraint (32g) is approximated as

(37) $$\xi_{k}\sigma^{2}\leq P_{m}\varpi \left(\psi_{i}^{\left[z\right]},\psi_{i},h_{m,k},h_{m,k}\right),\forall k\in K.$$

Therefore, utilizing the SCA method, the non-convex SINR expressions (32e)–(32g) are replaced by (36) and (37), which are convex. This approach can be applied directly to the other five transmission modes, so we simplify by omitting their discussion here.

- **Step 1.2: The penalty method for non-convex STAR RIS constraints**:

After Step 1.1, the optimization problems for passive beamforming in all six modes become convex except for the constraints related to the phase and amplitude of the STAR RIS. In the following, we specify the penalty methods proposed to handle these constraints for the *ES*, *MS*, and *TS* protocols.

*ES*: For constraint (25e), we adopt the penalty method proposed in [23]. By introducing a large positive constant *C*, the objective function is transformed into $t^{j}+C\sum_{n=1}^{N}\left(\right|\psi_{n,r}{|}^{2}+|\psi_{n,t}{|}^{2}-1),j\in \left\{\mathrm{FE},\mathrm{HE}\right\}$ with an additional constraint

(38) $$|\psi_{n,r}{|}^{2}+{\left|\psi_{n,t}\right|}^{2}\leq 1.$$

Using the first-order Taylor approximation of $\left(|\psi_{n,r}{|}^{2}+|\psi_{n,t}{|}^{2}\right)$ at iteration [z], we obtain the approximated objective function as

(39) $$t^{j}+C\sum_{n=1}^{N}ℜ\{2{\left(\psi_{n,r}^{\left[z\right]}\right)}^{*}\psi_{n,r}-|\psi_{n,r}^{\left[z\right]}{|}^{2}+2{\left(\psi_{n,t}^{\left[z\right]}\right)}^{*}\psi_{n,t}-|\psi_{n,t}^{\left[z\right]}{|}^{2}\}.$$

*MS*: For constraint (26b), it is obvious that the amplitude coefficient of each STAR RIS element in the *FM* and *HM* modes is an integer chosen from 0 to 1. To transform constraints (26b), it is worth noting that no matter whether each element is 0 or 1, it always satisfies (38) and

(40) $$-|\psi_{n,i}{|}^{2}+\left|\psi_{n,i}\right|=0,i\in \left\{r,t\right\}.$$

(40) forces the amplitudes of $\psi_{n,r}$ and $\psi_{n,t}$ to 0 or 1. Due to constraint (38), $0\;\leq |\psi_{n,i}|\leq \;1$, we obtain that

(41) $$-|\psi_{n,i}{|}^{2}+\left|\psi_{n,i}\right|\geq 0,i\in \left\{r,t\right\}.$$

Hence, constraint (26b) can be replaced by adding a penalty term to the objective function as $t^{j}-C\sum_{n=1}^{N}\left(-\right|\psi_{n,r}{|}^{2}+|\psi_{n,r}\left|-\right|\psi_{n,t}{|}^{2}+|\psi_{n,t}\left|\right),j\in \left\{\mathrm{FM},\mathrm{HM}\right\}$. Applying the first-order Taylor approximation to the penalty term $-|\psi_{n,r}{|}^{2}-{\left|\psi_{n,t}\right|}^{2}$ at iteration [z], the objective function is transformed to

(42) $$t^{j}-C\sum_{n=1}^{N}\left(A^{\left[z\right]}-2ℜ\left\{{\left(\psi_{n,r}^{\left[z\right]}\right)}^{*}\psi_{n,r}\right\}-2ℜ\left\{{\left(\psi_{n,t}^{\left[z\right]}\right)}^{*}\psi_{n,t}\right\}\right),$$

where $A^{\left[z\right]}={\left|\psi_{n,r}^{\left[z\right]}\right|}^{2}+{\left|\psi_{n,t}^{\left[z\right]}\right|}^{2}+|\psi_{n,r}|+|\psi_{n,t}|$.

*TS*: For *TS* modes, we also adopt the penalty method proposed in [23] to address constraints (27b), which transforms the objective function into $t^{j}+C\sum_{n=1}^{N}\left(\right|\psi_{n,r}{|}^{2}-1+|\psi_{n,t}{|}^{2}-1),j\in \left\{\mathrm{FT},\mathrm{HT}\right\}$ with an additional constraint

(43) $$|\psi_{n,i}{|}^{2}\leq 1,n\in N,i\in \left\{r,t\right\}.$$

The objective function is then approximated in the same way as (39).

After Steps 1.1 and 1.2, the STAR RIS optimization subproblems for all six transmission modes become convex quadratically constrained quadratic program (QCQP) and can be solved using the CVX optimization toolbox. The detailed process to solve the STAR RIS passive beamforming optimization problem is summarized in Algorithm 1.
**Algorithm 1:** STAR RIS passive beamforming optimization algorithm for six transmission modes
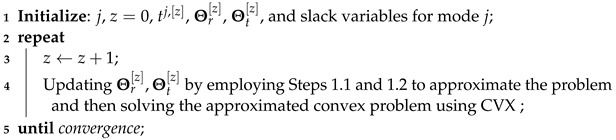


#### 3.1.2. Joint Optimization of the Active Beamforming, Common Rate and Time Allocation

Given $\Theta_{r}$ and $\Theta_{t}$, the effective channels of all users are fixed. To jointly optimize the remaining variables, we first introduce an auxiliary variable $x^{j}$ to denote the objective function for the transmission mode *j*, leading to the following constraints:

(44) $$R_{p,k}^{j}+C_{k}\geq x^{j},\forall k\in K,j\in \left\{\mathrm{FE},\mathrm{FM},\mathrm{FT},\mathrm{HE},\mathrm{HM},\mathrm{HT}\right\}.$$

In this subproblem, non-convexity arises from both the fractional SINR expressions and the coupling of time allocation variables within the rate expressions. Both of these challenges are tackled using SCA in the following steps.

- **Step 2.1: The SCA method for time allocation variables**:

In this step, we first specified the SCA methods employed to address the coupled time allocation variables within the rate expressions. Note that in the *FE* and *FM* modes, there are no time allocation variables. The non-convexity of the corresponding optimization problems arises solely from the SINR expressions, which are directly addressed in Step 2.2.

*FT*: To optimize $\lambda_{r}$ in problem (27), we first introduce the slack variables $\alpha^{i}={\left[\alpha_{1}^{i},\ldots ,\alpha_{K}^{i}\right]}^{T}$, and $\alpha_{c}^{i}={\left[\alpha_{c,1}^{i},\ldots ,\alpha_{c,K}^{i}\right]}^{T}$, i∈{r,t} to denote the private and common rates. This allows us to transform (44) and (25b) to

(45a)λrαkr+(1−λr)αkt+Ck≥xFT,(45b)λrαc,kr+(1−λr)αc,kt≥∑m∈KCm,(45c)αki≤log2(1+γkFD,i),(45d)αc,ki≤log2(1+γc,kFD,i).

For the bilinear function $\lambda_{r}\alpha_{k}^{r}$, it can be approximated by the first-order Taylor approximation at iteration [z] based on ν(·) in (34). Hence, constraints (45a) and (45b) are approximated as

(46a)−ν(αkr,[z],−λr)+αkt−ν(αkt,[z],−λr)+Ck≥xFT,(46b)−ν(αc,kr,[z],−λr)+αc,kt−ν(αc,kt,[z],−λr)≥∑m∈KCm.

*HE/HM*: To optimize λ in problems (28) and (29), akin to the *FT* mode, we introduce the slack variables α, and $\alpha_{c}^{i}$ to represent the private and common rates, thereby facilitating the transformation of (44) and (25b) to

(47a)λαk+Ck≥xj,(47b)λαc,k≥∑m∈KCm,k∈K1(47c)λαc,k+(1−λ)log(1+γc,kHD,[2])≥∑m∈KCm,k∈K2(47d)αk≤log2(1+γkHD),(47e)αc,k≤log2(1+γc,kHD,[1]).

Using the same first-order Taylor approximation method in (46), constraints (47a)–(47c) are approximated to

(48) $$\begin{array}{cc} \; & -\nu \left(\alpha_{k}^{\left[z\right]},-\lambda \right)+C_{k}\geq x^{j}, \end{array}$$

(49) $$\begin{array}{cc} \; & -\nu \left(\alpha_{c,k}^{\left[z\right]},-\lambda \right)\geq \sum_{m\in K}C_{m},\forall k\in K_{1}, \end{array}$$

(50) $$\begin{array}{cc} \; & -\nu \left(\alpha_{c,k}^{\left[z\right]},-\lambda \right)+\left(1-\lambda \right)\log_{2}\left(1+\gamma_{c,k}^{\mathrm{HD},[2]}\right)\geq \sum_{m\in K}C_{m},\forall k\in K_{2}. \end{array}$$

*HT*: To jointly optimize $\lambda_{r}$, $\lambda_{1}$, and $\lambda_{2}$ in problems (28), we also introduce the slack variables $\alpha^{i}$, and $\alpha_{c}^{i}$, i∈{r,t} to transform the problem. By applying the approximation method described in (46), we approximate (44) and (25b) to

(51a)−ν(αkr,[z],−λ1)−ν(αkt,[z],−λ2)+Ck≥xHT,(51b)−ν(αc,kr,[z],−λ1)−ν(αc,kt,[z],−λ2)≥∑m∈KCm,∀k∈K1, −ν(αc,kr,[z],−λ1)+(λr−λ1)log2(1+γc,kHD,[2],r)−(51c)ν(αc,kr,[z],−λ2)+(1−λr−λ2)log2(1+γc,kHD,[2],t)≥∑m∈KCm,∀k∈K2,(51d)αc,ki≥log(1+γc,kHD,[1],i),(51e)αki≥log(1+γkHD,i).

Following Step 2.1, the time allocation variables are decoupled from the non-convex SINR expressions for all transmission modes. Next, we proceed to address the classical non-convex SINR expressions.

- **Step 2.2: The SCA method for non-convex SINRs**:

Similar to Step 1.1, the SCA method for SINRs is applicable to all transmission modes. Therefore, we illustrate this approach using the *FE* problem (25) as an example, without detailing other modes.

We first introduce slack variables $\iota ={\left[\iota_{1},\cdots ,\iota_{K}\right]}^{T}$, $\iota_{c}={\left[\iota_{c,1},\cdots ,\iota_{c,K}\right]}^{T}$ to denote the SINRs of $s_{k}$ and $s_{0}$. Constraints (44) and (25b) are equivalently transformed to

(52a)log2(1+ιk)+Ck≥xFE,∀k∈K,(52b)log2(1+ιc,k)≥∑m∈KCm,k∈K,(52c)ιc,k≤γc,kFD,[1],∀k∈K,(52d)ιk≤γkFD,∀k∈K.

Constraints (52c) and (52d) remain non-convex, we then introduce variables $\zeta ={\left[\zeta_{1},\cdots ,\zeta_{K}\right]}^{T}$, $\zeta_{c}={\left[\zeta_{c,1},\cdots ,\zeta_{c,K}\right]}^{T}$ to, respectively, denote the denominators of SINRs for $s_{k}$ and $s_{0}$. (52c) and (52d) are equivalently transformed to

(53a)ζc,k≥de(γc,kFD,[1]),∀k∈K,(53b)ζk≥de(γkFD),∀k∈K,(53c)ιc,kζc,k≤g˜i,kHp02,(53d)ιkζk≤g˜i,kHpk2.

Following the approaches specified in Step 1.1 to address the non-convexity in (33), we approximate constraints (53c) and (53d) at iteration [z] to

(54a)ν(ιc,k[z],ζc,k[z])≤ϖ(p0[z],p0,0,g˜i,k),∀k∈K,(54b)ν(ιk[z],ζk[z])≤ϖ(pk[z],pk,0,g˜i,k),∀k∈K.

Based on the approximation methods specified in Steps 2.1 and 2.2, the joint active beamforming, common rate, and time allocation optimization problems for all six transmission modes become QCQP and can be solved using CVX. The detailed process of the SCA methods to solve this subproblem is illustrated in Algorithm 2.
**Algorithm 2:** Joint transmit active beamforming and resource allocation algorithm
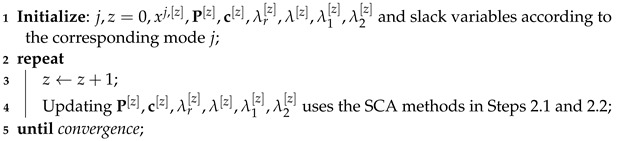


#### 3.1.3. Alternative Optimization

Based on Algorithms 1 and 2, the proposed AO algorithm that solves the two subproblems iteratively is shown in Algorithm 3. Starting from a feasible point $\left(P^{\left[0\right]},\Theta_{r}^{\left[0\right]},\Theta_{t}^{\left[0\right]},c^{\left[0\right]},\lambda_{r}^{\left[0\right]},\lambda^{\left[0\right]},\lambda_{1}^{\left[0\right]},\lambda_{2}^{\left[0\right]}\right)$, at each iteration [z], we first update $\Theta_{r}^{\left[z\right]}$ and $\Theta_{t}^{\left[z\right]}$ by Algorithm 1. Given $\Theta_{r}^{\left[z\right]}$ and $\Theta_{t}^{\left[z\right]}$, we then update $P^{\left[z\right]}$, the common rate and time allocation based on Algorithm 2. By iteratively solving these two subproblems, the objective function is updated until convergence.
**Algorithm 3:** Proposed AO algorithm
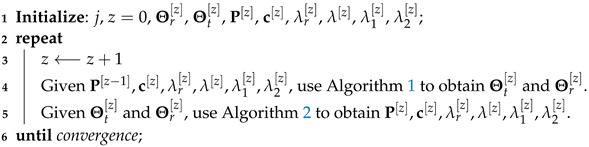


### 3.2. Proposed Low-Complexity Algorithm

In this subsection, we propose a low-complexity algorithm for the STAR RIS passive beamforming and transmit beamforming optimization subproblems.

#### 3.2.1. STAR RIS Passive Beamforming Optimization

We begin by specifying the proposed low-complexity algorithm tailored for the *ES* protocol. Subsequently, we extend this algorithm to the *MS* and *TS* protocols.

*ES*: To develop a low-complexity algorithm for designing $\Theta_{r}$ and $\Theta_{t}$ in the ES protocol, we consider the following problem aimed at maximizing the sum of channel gains:

(55) $$\begin{array}{cc} \max_{\Theta_{r},\Theta_{t}}\;\; & \sum_{k=1}^{K}∥g_{k}^{H}+h_{k}^{H}\Theta_{i}{E∥}^{2},i\in \left\{r,t\right\}\\ s.t.\;\; & (25c),\;(25e). \end{array}$$

Constraints (25c) and (25e) are non-convex, which make the problem (55) difficult to solve. To tackle such a non-convex problem, we first relax constraints (25c) and (25e) to $∥\Theta_{r}{∥}_{F}^{2}+{∥\Theta_{t}∥}_{F}^{2}\leq N$, where ${∥\cdot ∥}_{F}$ denotes the Frobenius norm. This approximation relaxes the feasible solutions of $\Theta_{r}$ and $\Theta_{t}$ to a convex set $S=\{\left(\Theta_{r},\Theta_{r}\right)|∥\Theta_{r}{∥}_{F}^{2}+∥\Theta_{t}{∥}_{F}^{2}\leq N\}$. Therefore, problem (55) is relaxed to

(56a)maxΘf(Θ)≜∥GH+HHΘEx∥F2(56b)s.t.Θ∈S,

where $\Theta =\left[\begin{array}{cc} \Theta_{r} & 0\\ 0 & \Theta_{t} \end{array}\right]$, $G=\left[G_{r};G_{t}\right],G_{r}=\left\{g_{k}|k\in K_{r}\right\},G_{t}=\left\{g_{k}|k\in K_{t}\right\}$, $H=\left[H_{r};H_{t}\right],H_{r}=\left\{h_{k}|k\in K_{r}\right\},H_{t}=\left\{h_{k}|k\in K_{t}\right\}$ and $E_{x}=\left[E;E\right]$.

Inspired by the method proposed in [24], we employ the gradient decent method at the point $\Theta_{0}=0$ to derive a closed-form solution of (56). The corresponding gradient of f(Θ) at $\Theta_{0}=0$ is

(57) $${▽}_{\Theta }f\left(\Theta_{0}\right)=HG^{H}E_{x}^{H},$$

By using (57) as the decent direction, i.e., $D_{0}={▽}_{\Theta }f\left(\Theta_{0}\right)$, we then take the Armijo rule to determine the step size α as

(58) $$f\left(\Theta_{0}+\alpha D_{0}\right)\geq f\left(\Theta_{0}\right)+\phi \cdot \alpha \cdot \mathrm{tr}\left({▽}_{\Theta }f{\left(\Theta_{0}\right)}^{H}D_{0}\right),$$

where ϕ∈(0,0.5) is a constant shrinkage factor. Substituting (57) into (58), we obtain

(59) $$\alpha^{2}∥H^{H}HG^{H}E_{x}^{H}E_{x}{∥}_{F}^{2}+\alpha \cdot \left(2-\phi \right){∥HG^{H}E_{x}^{H}∥}_{F}^{2}\geq 0.$$

To guarantee the solution is on the boundary of the convex set S, we set $\alpha =\frac{\sqrt{N}}{∥HG^{H}E_{x}^{H}{∥}_{F}}$. Then, we obtain the following solution of problem (56) as

(60) $$\Theta =\frac{\sqrt{N}}{∥HG^{H}E_{x}^{H}{∥}_{F}}HG^{H}E_{x}^{H}.$$

However, the solution (60) for the relaxed problem (56) may not satisfy the original constraints (25c), (25e). Therefore, we propose to project Θ into the feasible region. Following [24], we first define the operator sym(·) that projects any square matrix X to a symmetric matrix, i.e.,

(61) $$\mathrm{sym}\left(X\right)=\frac{1}{2}\left(X^{T}+X\right),$$

and the operator uni(·) which projects an arbitrary matrix X to a unitary matrix as

(62) $$\mathrm{uni}\left(X\right)={\mathrm{UV}}^{H},$$

where U and V are unitary matrices obtained by the singular value decomposition (SVD) of X, i.e., $X={\mathrm{USV}}^{H}$. Consider X with dimension *N* and rank *R*. Then, the unitary matrices U and V can be partitioned as $U=[U_{R},U_{N-R}]$ and $V=[V_{R},V_{N-R}]$, respectively. Based on the operators in (61) and (62), we could then define the symmetric unitary projection as [24]

(63) $$\mathrm{symuni}\left(X\right)=\mathrm{uni}\left(\mathrm{sym}\left(X\right)\right)=\hat{U}V^{H},$$

where $\hat{U}=\left[U_{R},V_{N-R}^{*}\right]$.

Utilizing the symmetric unitary projection, we then derive a feasible STAR RIS passive beamforming solution Θ. It is noteworthy that both $\Theta_{r}$ and $\Theta_{t}$ are diagonal matrices, implying that Θ is also diagonal. The diagonal entries of Θ are determined by

(64) $$\mathrm{diag}\left\{\Theta \right\}=\frac{\sqrt{2}}{2}\mathrm{symuni}\left\{\mathrm{diag}\left(HG^{H}E_{x}^{H}\right)\right\}.$$

Since the unitary matrix $\mathrm{symuni}\left\{\mathrm{diag}\left(HG^{H}E_{x}^{H}\right)\right\}\in C^{2N\times 2N}$ whose dimension is 2N, we have $∥\mathrm{symuni}\left\{\mathrm{diag}\left(HG^{H}E_{x}^{H}\right)\right\}{∥}_{F}^{2}=2N$. To ensure constraint (25e), we scale each entry with $\frac{\sqrt{2}}{2}$.

*MS*: For the *MS* mode, we first utilize (64) to derive $\Theta_{r}$ and $\Theta_{t}$. As each element can only operate in either reflection or transmission mode, we then compare the reflection and transmission amplitude coefficients of each STAR RIS element. The element with the higher coefficient is designated as 1, while the one with the lower coefficient is designated as 0. Based on (64), the reflection and transmission coefficients of each STAR RIS element are designed as

(65) $$\begin{array}{cc} \beta_{r,n} & =\left\{\begin{array}{c} 1,\;\mathrm{if}\;|\Theta_{n,n}\left|\;-\;\right|\Theta_{N+n,N+n}|\;\geq 0\\ 0,\;\mathrm{if}\;|\Theta_{n,n}\left|\;-\;\right|\Theta_{N+n,N+n}|\;<0 \end{array}\right.,\;\forall n\in N\\ \beta_{t,n} & =1-\beta_{r,n}. \end{array}$$

*TS*: For the *TS* mode, in the reflection time slots, $\Theta_{t}=0$, the amplitudes of all elements in $\Theta_{r}$ are 1. The solution of the reflection matrix is given as

(66) $$\Theta_{r}=\mathrm{symuni}\left\{\mathrm{diag}\left(H_{r}G_{r}^{H}E^{H}\right)\right\}.$$

In the transmission time slots, the transmission matrix is given as

(67) $$\Theta_{t}=\mathrm{symuni}\left\{\mathrm{diag}\left(H_{t}G_{t}^{H}E^{H}\right)\right\}.$$

#### 3.2.2. Joint Optimization of the Active Beamforming, Common Rate and Time Allocation

With fixed $\Theta_{r}$ and $\Theta_{t}$, we aim to simplify the design of the remaining variables. To achieve this, we reduce the optimization dimension by fixing the direction of the precoders. Subsequently, we focus on jointly optimizing the power, common rate, and time allocation variables.

Let ${\bar{g}}_{k}=g_{k}/\left|\right|g_{k}\left|\right|$, $\bar{G}=\left[{\bar{g}}_{1},\ldots ,{\bar{g}}_{K}\right]$, ${\bar{p}}_{k}=p_{k}/\left|\right|p_{k}\left|\right|$, and $\bar{P}=\left[{\bar{p}}_{1},\ldots ,{\bar{p}}_{K}\right]$. We transform the active beamformings in the form of $p_{c}=\sqrt{\rho_{c}P_{t}}{\bar{p}}_{c},p_{k}=\sqrt{\rho_{k}P_{t}}{\bar{p}}_{k}$, where $\rho_{c}$ and $\rho_{k}$ are the power allocation coefficients. For the beamforming direction ${\bar{p}}_{k}$ of the private streams, we adopt zero-forcing (ZF) beamforming, i.e., $\bar{P}=\bar{G}{\left({\bar{G}}^{H}\bar{G}\right)}^{-1}$ [25]. For the beamforming direction ${\bar{p}}_{c}$ of the common stream, we utilize maximum ratio transmission (MRT) and SVD methods as ${\bar{p}}_{c}=u_{c}$, where $u_{c}=U_{c}\left(:,1\right),G=U_{c}S_{c}V_{c}$ [2]. Based on the aforementioned beamforming direction design, the optimization problems for all six transmission modes are simplified. In the following, we illustrate this simplification by considering the *FD* mode as a representative example:

(68a)maxρ,cmink∈KCk+Rp,kFE(68b)s.t.∑m∈KCm≤Rc,kFE,∀k∈K,(68c)ρc+∑k∈Kρk≤1,

where $\rho =[\rho_{c},\rho_{1},\ldots ,\rho_{K}]$ are the power allocation coefficients of the BS, we have $R_{p,k}^{\mathrm{FE}}=\log_{2}\left(1+\rho_{k}P_{t}{\left|{\tilde{g}}_{k}^{H}{\bar{p}}_{k}\right|}^{2}/\sigma^{2}\right)$, $R_{c,k}^{\mathrm{FE}}=\log_{2}\left(1+\rho_{c}P_{t}|{\tilde{g}}_{k}^{H}{\bar{p}}_{c}{|}^{2}/(\rho_{k}P_{t}|{\tilde{g}}_{k}^{H}{\bar{p}}_{k}{|}^{2}+\sigma^{2})\right)$. The optimization problem can be directly solved using the SCA-based approach specified in Algorithm 2. The optimization approach for other transmission modes aligns with that of the FE mode. For *FT/HE/HM/HT* modes, the additional time allocation variables are jointly optimized using the SCA-based approach specified in Step 2.1.

The detailed process of the proposed low-complexity algorithm to solve problems (25)–(30) is outlined in Algorithm 4. Contrasted with Algorithm 3, the primary difference lies in the closed-form STAR RIS passive beamforming design and the closed-form active beamforming direction design. Additionally, there is no alternating optimization between the two subproblems. Each subproblem is solved once before the output.
**Algorithm 4:** Proposed low-complexity algorithm**1** **Initialize**: $j,P^{\left[0\right]},c^{\left[0\right]},\lambda_{r}^{\left[0\right]},\lambda^{\left[0\right]},\lambda_{1}^{\left[0\right]},\lambda_{2}^{\left[0\right]}$**2** Update $\Theta_{t}$ and $\Theta_{r}$ through (64) if j=FE/HE, (65) if j=FM/HM, (66) and (67) if j=FT/HT.**3** Given $\Theta_{t}$ and $\Theta_{r}$, update P, c, $\lambda_{r}$, λ, $\lambda_{1}$, $\lambda_{2}$ by solving the joint power, common rate, and time allocation problem via Algorithm 2.

### 3.3. Convergence and Computational Complexity Analysis

#### 3.3.1. Convergence Analysis

In Algorithm 1, the SCA method guarantees the objective $t^{j}$ monotonically increases, meaning that $t^{j,[z-1]}\leq t^{j,[z]}$. This arises from the fact that the solution of $\Theta_{r}$ and $\Theta_{t}$ at iteration [z−1] is a feasible point of the STAR RIS problem at iteration [z]. Due to STAR RIS power constraint (25e), there is an upper bound for $t^{j,[z]}$. Hence, the convergence of Algorithm 1 is guaranteed. Similarly, in Algorithm 2, the SCA method guarantees that $x^{j,[z-1]}\leq x^{j,[z]}$ since the solution at iteration [z−1] is an also feasible point of the joint optimization problem at iteration [z]. Due to the power constraint (25d) at the BS, there is an upper bound for the objective value. Hence, the convergence of the Algorithm 2 is guaranteed. The convergence of Algorithms 1 and 2 implies that the objective of the AO algorithm increases monotonically, which guarantees the convergence of Algorithm 3.

#### 3.3.2. Computational Complexity of Algorithm 3

The computational complexity of Algorithm 1 is $O\left(N^{3.5}\log_{2}\left(1/\epsilon_{1}\right)\right)$, where $\epsilon_{1}$ is the convergence tolerance. The complexity of Algorithm 2 is $O\left({\left(KN_{t}\right)}^{3.5}\log_{2}\left(1/\epsilon_{2}\right)\right)$, where $\epsilon_{2}$ is the corresponding convergence tolerance. Therefore, the computational complexity of Algorithm 3 is $O\left(T\left(N^{3.5}\log_{2}\left(1/\epsilon_{1}\right)+{\left(KN_{t}\right)}^{3.5}\log_{2}\left(1/\epsilon_{2}\right)\right)\right)$, where *T* denotes the number of iterations of the AO algorithm.

#### 3.3.3. Computational Complexity of Algorithm 4

For the proposed low-complexity algorithm, the computational complexity of the STAR RIS passive beamforming design is mainly occupied by the unitary projection with the SVD method, which is $O\left(N^{3}\right)$. For the active beamforming optimization subproblem, the computational complexity is $O\left(K^{3.5}\log_{2}\left(1/\epsilon_{2}\right)\right)$. Therefore, the overall computational complexity of Algorithm 4 is $O\left(N^{3}+K^{3.5}\log_{2}\left(1/\epsilon_{2}\right)\right)$, which is significantly lower than that of Algorithm 3.

### 3.4. Imperfect CSIT

Due to quantization errors in CSI feedback, feedback delays, and many other issues, it is difficult for the BS to obtain the perfect CSIT. Hence, in this subsection, we focus on an imperfect CSIT scenario where the direct channel between the BS and users, the RIS and users is imperfect. Since the locations of the BS and STAR RIS are typically fixed, perfect CSIT between the BS and the STAR RIS is considered. In order to simplify the notations, we introduce a block diagonal matrix Q including all perfect channel coefficients, which is given as

(69) $$Q=\mathrm{diag}\left(\left[g_{1},\ldots ,g_{K}\right],\left[h_{1},\ldots ,h_{K}\right],E\right).$$

We denote the estimated CSI between the BS and user-*k* as ${\hat{g}}_{k}$, and the estimated CSI between the STAR RIS and user-*k* as ${\hat{h}}_{k}$. Besides, we denote ${\tilde{g}}_{k}$ and ${\tilde{h}}_{k}$ as the corresponding estimation errors, both following independent and identically distributed (i.i.d) zero-mean complex Gaussian distributions. The corresponding error covariance matrices are denoted as $E_{g_{k}|{\hat{g}}_{k}}\left\{g_{k}g_{k}^{H}|{\hat{g}}_{k}\right\}=R_{g,k}$ and $E_{h_{k}|{\hat{h}}_{k}}\left\{h_{k}h_{k}^{H}|{\hat{h}}_{k}\right\}=R_{h,k}$, respectively. The relationship between the real channel matrix Q and the estimated channel matrix $\hat{Q}$ is modeled as

(70) $$Q=\hat{Q}+\tilde{Q},$$

where $\hat{Q}=\mathrm{diag}\left(\left[{\hat{g}}_{1},\ldots ,{\hat{g}}_{K}\right],\left[{\hat{h}}_{1},\ldots ,{\hat{h}}_{K}\right],E\right)$ and $\tilde{Q}=\mathrm{diag}\left(\left[{\tilde{g}}_{1},\ldots ,{\tilde{g}}_{K}\right],\left[{\tilde{h}}_{1},\ldots ,{\tilde{h}}_{K}\right],0\right)$.

With partial CSIT, the instantaneous rate cannot guarantee decodability for the users. A more robust approach is to optimize the ER which characterizes the long-term rate performance across all channel states. The ERs for the common and private streams at user-*k* are defined as

(71a)R¯c,kj=E{Q^,Q}Rc,kj,(71b)R¯p,kj=E{Q^,Q}Rp,kj.

Optimizing ERs directly is very challenging. Instead, we can leverage average rates (ARs) to obtain ERs [26]. With a given channel estimation $\hat{Q}$, ARs for the common and private streams at user-*k* are defined as

(72a)R^c,kj=E{Q|Q˜}Rc,kj|Q^,(72b)R^p,kj=E{Q|Q˜}Rp,kj|Q^.

The relation between ERs and ARs is as follows [26]

(73a)R¯c,kj=EQ^R^c,kj,(73b)R¯p,kj=EQ^R^p,kj.

To ensure that the common message $s_{0}$ is successfully decoded by all users, the ER of the common stream $s_{0}$ should satisfy

(74) $$\begin{array}{c} {\bar{R}}_{c}^{j}=\min_{k\in K}\left\{{\bar{R}}_{c,k}^{j}\right\}. \end{array}$$

By replacing the instantaneous rates in (25)–(30) with the average rates in (72), we obtain the stochastic AR optimization problem for a given channel estimation $\hat{Q}$. A conventional approach to address such stochastic problems is the sampled average approximation (SAA) approach [2], which transforms the problems into deterministic ones. However, such an approach requires a large number of channel samples and significantly increases the computational complexity. To avoid these issues, we follow [27] and obtain the lower bounds for the ARs. For example, the AR ${\hat{R}}_{c,k}^{j}$ in (72) is bounded by

(75) $$\begin{array}{cc} {\hat{R}}_{c,k}^{j}\; & =E_{Q|\hat{Q}}\left\{\log_{2}\left(1+\frac{|g_{k}^{H}p_{c}+h_{k}^{H}\Theta_{i}Ep_{c}{|}^{2}}{\sum_{j=1}^{K}{\left|g_{k}^{H}p_{j}+h_{k}^{H}\Theta_{i}Ep_{j}\right|}^{2}+\sigma_{k}^{2}}\right)\right\}\\ \; & \geq \log_{2}\left(1+\frac{|{\hat{g}}_{k}^{H}p_{c}+{\hat{h}}_{k}^{H}\Theta_{i}Ep_{c}{|}^{2}}{\sum_{j=1}^{K}\left(|{\hat{g}}_{k}^{H}p_{j}{|}^{2}+p_{j}^{H}R_{g,k}p_{j}+q_{i,j}^{H}R_{h,k}q_{i,j}\right)+p_{c}^{H}R_{h,k}p_{c}+q_{i,c}^{H}R_{h,k}q_{i,c}+\sigma_{k}^{2}}\right),\\ \; & ≜{\hat{R}}_{c,k}^{j,lb}, \end{array}$$

where $q_{i,j}=\Theta_{i}Ep_{j}$. The inequality is obtained by treating $e_{g,k}^{H}p_{c}s_{c}+e_{h,k}^{H}q_{i,c}s_{c}+\sum_{j=1}^{K}\left(e_{k}^{H}p_{j}+e_{h,k}^{H}q_{i,j}\right)s_{j}$ as independent Gaussian noise and applying Jensen’s inequality [28]. Similarly, the AR ${\hat{R}}_{p,k}^{j}$ is bounded by

(76) $$\begin{array}{cc} {\hat{R}}_{p,k}^{j}\; & =E_{Q|\hat{Q}}\left\{\log_{2}\left(1+\frac{|g_{k}^{H}p_{k}+h_{k}^{H}\Theta_{i}Ep_{k}{|}^{2}}{\sum_{j\neq k}^{K}{\left|g_{k}^{H}p_{j}+h_{k}^{H}\Theta_{i}Ep_{j}\right|}^{2}+\sigma_{k}^{2}}\right)\right\}\\ \; & \geq \log_{2}\left(1+\frac{|{\hat{g}}_{k}^{H}p_{k}+{\hat{h}}_{k}^{H}\Theta_{i}Ep_{k}{|}^{2}}{\sum_{j\neq k}^{K}{\left|{\hat{g}}_{k}^{H}p_{j}\right|}^{2}+\sum_{j=1}^{K}p_{j}^{H}R_{g,k}p_{j}+\sum_{j=1}^{K}q_{i,j}^{H}R_{h,k}q_{i,j}+\sigma_{k}^{2}}\right)\\ \; & ≜{\hat{R}}_{p,k}^{j,lb}. \end{array}$$

With the lower bound (75), the ER of the common stream is, therefore, bounded by

(77) $$\begin{array}{c} {\bar{R}}_{c}=\min_{k\in K}\left\{E_{\hat{Q}}\left\{{\hat{R}}_{c,k}^{j}\right\}\right\}\geq \min_{k\in K}\left\{E_{\hat{Q}}\left\{{\hat{R}}_{c,k}^{j,lb}\right\}\right\}\geq E_{\hat{Q}}\left\{\min_{k\in K}\left\{{\hat{R}}_{c,k}^{j,lb}\right\}\right\}. \end{array}$$

Similarly, the ER of the private stream is bounded by

(78) $$\begin{array}{c} {\bar{R}}_{p}=E_{\hat{Q}}\left\{{\hat{R}}_{p,k}^{j}\right\}\geq E_{\hat{Q}}\left\{{\hat{R}}_{p,k}^{j,lb}\right\}. \end{array}$$

By replacing the instantaneous rates $R_{c}^{j}$ and $R_{p,k}^{j}$ in (25)–(30) with the lower bounds $\min_{k\in K}{\hat{R}}_{c,k}^{lb}$ and ${\hat{R}}_{p,k}^{lb}$ in (75) and (76), we construct six long-term deterministic imperfect CSIT optimization problems. As the non-convexity in these problems remains the same as the perfect CSIT ones, our proposed two algorithms can be employed to address the imperfect CSIT optimization problems directly. Therefore, we omit the algorithm framework here.

## 4. Numerical Results

In this section, we evaluate the performance of our proposed STAR RIS-assisted CRS system framework along with the two proposed algorithms.

### 4.1. Simulation Setting

The setting of the simulation follows [29]. Consider a three-dimensional (3D) space, the BS is located at (0,0,0) m, the STAR RIS is located at (0,50,0) m. Users are randomly distributed within a circle, with the center being the location of the STAR RIS and a radius of r=5 m. We follow the method in [4] to separate the users into group $K_{1}$ and $K_{2}$. We only choose one user with the largest channel gain as the relaying user, other users are destination users. The channels between BS and users, as well as between user-*m* and user-*n* are assumed to be Rayleigh fading, and are denoted as $g_{k}=L_{\mathrm{BU},k}{\bar{g}}_{k},h_{m,n}=L_{\mathrm{UU}}{\bar{h}}_{m,n}$, where $L_{\mathrm{BU},k}$ and $L_{\mathrm{UU}}$ denote the path loss between the BS and users as well as the path loss between users. ${\bar{g}}_{k}$ and ${\bar{h}}_{m,n}$ follow the complex Gaussian distribution with a certain variance, i.e., ${\bar{g}}_{k}∼\mathrm{CN}\left(0,\sigma_{k}^{2}\right),{\bar{h}}_{m,n}∼\mathrm{CN}\left(0,\sigma_{m,n}^{2}\right)$. The channels between the BS and STAR RIS, as well as between STAR RIS and users are assumed to follow the Rician fading model with the Rician factor being 3 dB. The path loss for all the aforementioned channels is defined as $L_{x}=L_{0}{\left(d_{x}/d_{0}\right)}^{-\alpha_{x}},x\in \left\{\mathrm{BU},\mathrm{UU},\mathrm{BR},\mathrm{RU}\right\}$, where $d_{0}=1$ m is the reference distance, $L_{0}=-30$ dB is the path loss at reference distance, $d_{x}$ is the distance of different channels, and $\alpha_{x}$ denotes different channel path exponents. The channel path loss exponents between the BS and STAR RIS, as well as between STAR RIS and users are 2.2 [14]. The channel path loss exponent between the BS and users is 3.76 [23]. Besides, the relaying power is $P_{k}=0.5P_{t}$, the convergence tolerance is $\epsilon =10^{-3}$, and the noise power is σ=−90 dBm. All results are obtained by averaging over 100 random channel realizations. The variances of user channels are set to $\sigma_{k}^{2}=1$ for the relaying user, $\sigma_{k}^{2}=0.3$ for the destination users, and $\sigma_{m,n}^{2}=1$.

The following schemes are compared in this section:

- **CRS-FE/FM/FT/HE/HM/HT**: This refers to the STAR RIS-assisted CRS for six different transmission modes, as we proposed in Section 2.
- **CRS-HD/FD**: This refers to the existing CRS transmission schemes based on the *HD* or *FD* protocols without the assistance of STAR RIS [4,6].
- **RSMA-ES**: This refers to the existing STAR RIS-assisted RSMA transmission scheme without user relaying [16]. Here we only consider the *ES* protocol since it is more general than the *MS* and *TS* protocols.
- **SDMA-ES**: This refers to the existing STAR RIS-assisted SDMA transmission scheme. Again, we only consider the *ES* protocol.
- **NOMA-ES**: This refers to the existing STAR RIS assisted NOMA transmission scheme [29] with the *ES* protocol.
- **RSMA**: This refers to the conventional one-layer RSMA transmission scheme, which does not involve user relaying or assistance from STAR RIS [2].

### 4.2. Simulation Results

#### 4.2.1. Comparison Among STAR RIS Operating Protocols

**Figure 3:**
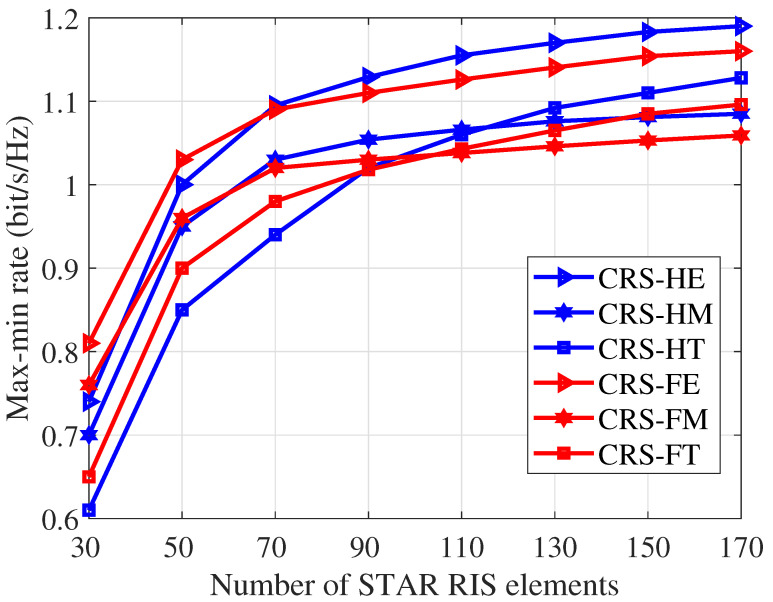
Max-min rate versus the number of STAR RIS elements *N*, SNR=20dB, K=4, $N_{t}=4$.

From Figure 3, we observe that the *FE/HE* mode consistently achieves a higher max-min rate than the *FM/HM* and *FT/HT* modes. This is because the *ES* protocol is more general than the *MS* and *TS* protocol, and it ensures that each STAR RIS element has a larger tunable range of the amplitude coefficient. As the number of STAR RIS elements increases, the max-min rate performance gain between *FM/HM* and *FT/HT* modes decreases. Surprisingly, when *N* is larger than 110, the *FT/HT* mode achieves a higher max-min rate than the *FM/HM* mode. This indicates that the *TS* protocol outperforms the *MS* protocol when the STAR RIS element is large. With an increasing number of STAR RIS elements, the performance improvement gained from having all elements operating within the same transmission/reflection space is outweighed by the performance degradation caused by partial time service.

Due to the superior max-min rate performance of *ES* compared to *MS* and *TS*, in the following simulation results, we only illustrate the proposed STAR RIS-assisted CRS in *FE* and *HE* modes for clarity.

#### 4.2.2. Comparison Between FD and HD Relaying Protocols

**Figure 4:**
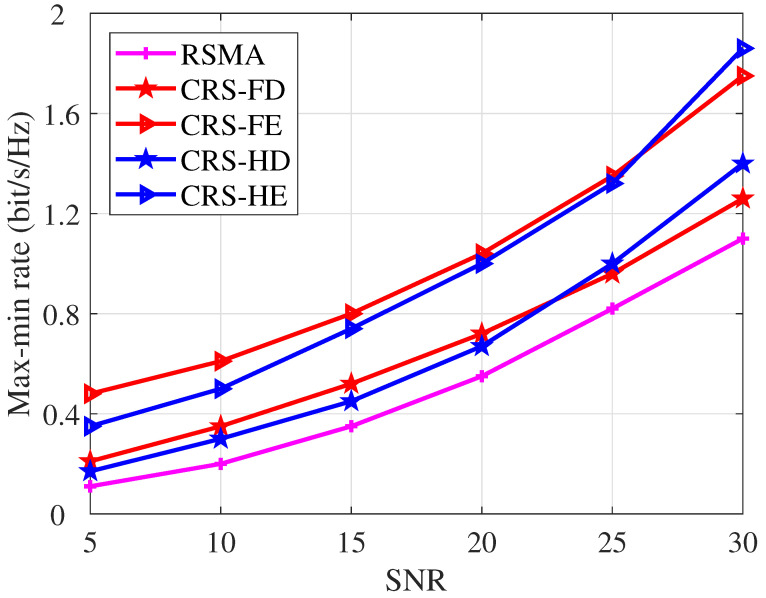
Max-min rate versus SNR, N=50, K=4, $N_{t}=4$.

Figure 4 illustrates the max-min rate performance versus transmit SNR. We compare our proposed CRS-FE/HE with the conventional RSMA scheme and the conventional CRS schemes with *HD* and *FD* protocols. Algorithm 3 is employed to optimize the corresponding problems of the proposed schemes. We set $N=50,K=4,N_{t}=4$. The max-min rate increases with SNR for all transmission schemes. It is noteworthy that when SNR is below 25 dB, the CRS-FE scheme achieves a higher max-min rate than the CRS-HE scheme. Conversely, when the SNR exceeds 25 dB, the CRS-HE scheme outperforms the CRS-FE scheme. This divergence stems from the introduction of self-interference in *FD* relaying. As the SNR increases, the level of self-interference also rises. This indicates that the *HD* protocol is preferred in the high SNR regime while the *FD* protocol is preferred in the low and moderate SNR regimes. For consistently ensuring the optimal max-min rate performance gain, we can employ *FD* relaying at low and moderate SNR and *HD* relaying at high SNR.

From Figure 4, it is obvious that with the assistance of STAR RIS and CRS, CRS-FE/HE achieves explicit max-min rate gain over conventional schemes. Specifically, CRS-FE demonstrates an average relative performance gain of 117.1% and 52.4% over conventional RSMA and CRS-FD, respectively. This demonstrates the significant performance enhancement achieved by integrating STAR RIS and CRS.

#### 4.2.3. Comparison Among Different MA Schemes

**Figure 5:**
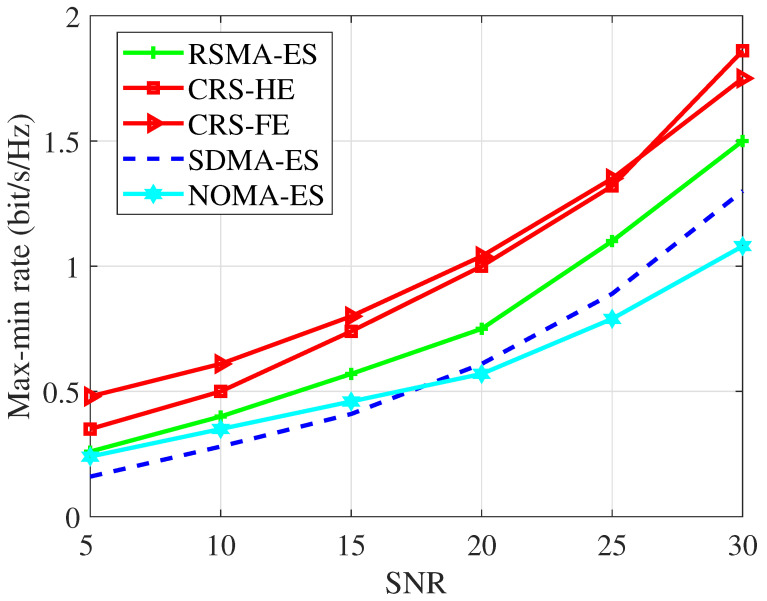
Max-minrate versus SNR, N=50, K=4, $N_{t}=4$.

In Figure 5, we compare various STAR RIS-assisted transmission schemes with the *ES* protocol when N=50, K=4, $N_{t}=4$. The results show that our proposed STAR RIS-aided CRS scheme outperforms other MA schemes. It achieves an average max-min rate gain of 42.6% over the RSMA-ES scheme, 90.9% over the SDMA-ES scheme, and 77.2% over the NOMA-ES scheme. Besides, the RSMA-ES scheme outperforms NOMA-ES and SDMA-ES. By splitting the user message into common and private parts, RSMA facilitates more flexible interference management in all SNR regimes.

#### 4.2.4. Comparison Between the Two Proposed Algorithms

**Figure 6:**
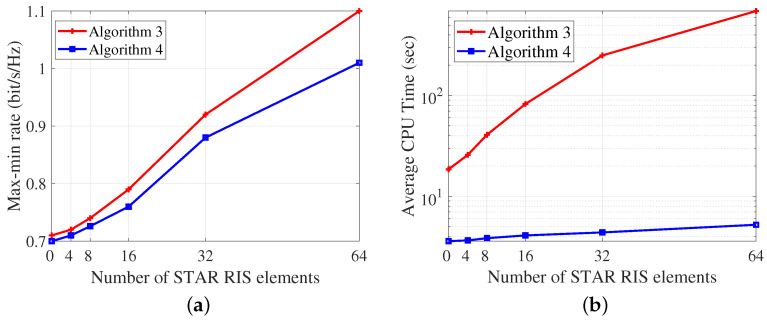
The performance of Algorithm 3 and 4. (**a**) Max-min rate versus *N*. (**b**) Average CPU time versus *N*.

In Figure 6, we compare the two proposed optimization algorithms for the proposed STAR RIS-aided CRS in the *ES* mode. We set SNR=20 dB, K=4, $N_{t}=4$. In comparison to the proposed AO algorithm, the low-complexity algorithm reduces the average CPU time by 99.2% while incurring only a 9.1% performance loss at N=64. This confirms the effectiveness of the proposed low-complexity algorithm. This efficiency is primarily due to the low-complexity algorithm eliminating the need for CVX operations to update $\Theta_{r}$ and $\Theta_{t}$, along with the alternative optimization between two subproblems. Instead, CVX is exclusively used for joint power, common rate, and time allocation, leading to a significant reduction in the optimization dimension. The results suggest that if the system can accommodate high time complexity, Algorithm 3 should be employed to achieve near-optimal max-min rate performance. Conversely, if the system is time-sensitive, the low-complexity Algorithm is recommended.

#### 4.2.5. Imperfect CSIT

**Figure 7:**
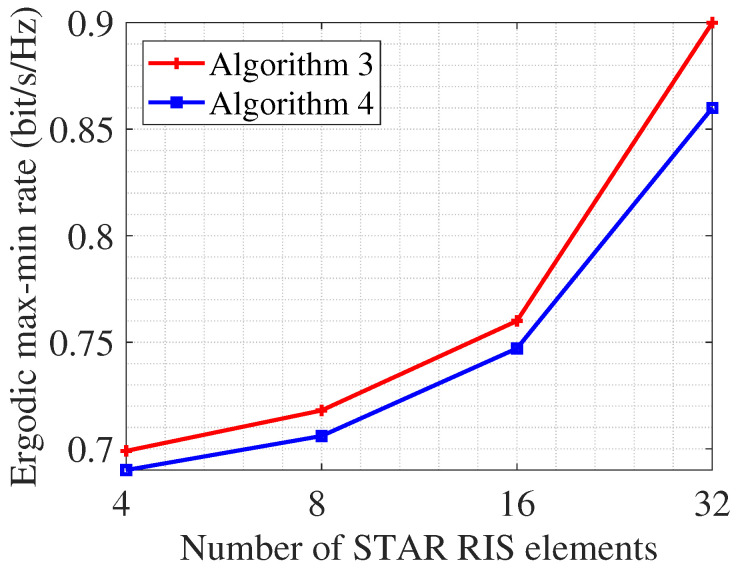
Ergodic max-min rate versus the number of STAR RIS elements *N*, SNR=20dB, K=4, $N_{t}=4$, κ=0.2.

Figure 7 illustrates the ergodic max-min rate versus the number of STAR RIS elements, where SNR=20 dB, K=4, $N_{t}=4$. The estimation errors $e_{g,k}$ and $e_{h,k}$ follow $\mathrm{CN}(0,\kappa \sigma_{k}^{2}I)$, where $\sigma_{k}^{2}$ represents the channel variance and κ represents the CSIT error variance ratio. In this work, we set κ=0.2. As the number of STAR RIS elements increases, the ergodic max-min rate increases accordingly. Our proposed low-complexity algorithm achieves performance close to that of the AO algorithm. This observation is consistent with our findings under perfect CSIT.

## 5. Conclusions

In this paper, we propose a novel STAR RIS-assisted CRS transmission with six different transmission modes, namely, *HE*, *HM*, *HT*, *FE*, *FM*, and *FT*. With the objective of maximizing the minimum user rate, we then propose a unified SCA-based AO algorithm to optimize the BS active beamforming and the STAR RIS passive beamforming iteratively under the transmit power constraint at the BS and the law of energy conservation at the STAR RIS. Meanwhile, we propose a novel low-complexity resource allocation algorithm that designs the BS active beamforming and the STAR RIS passive beamforming in closed form. Numerical results show that our proposed STAR RIS aid-CRS system achieves superior max-min rate performance gain over the existing CRS schemes and the STAR RIS-aided MA schemes. Furthermore, we show that the *FD* relaying has better max-min rate performance at low and moderate SNR while the *HD* relaying is preferred at high SNR. The *ES* protocol offers superior max-min rate performance compared to the *MS* and *TS* protocols, albeit with increased hardware complexity. With a large number of STAR RIS elements, the *TS* protocol demonstrates a significantly higher max-min rate gain compared to the *MS* protocol. Our proposed AO algorithm is better suited for systems that can accommodate higher time complexity, while the low-complexity algorithm is preferable for systems that are more time-sensitive.

## Data Availability

The data represented in this study are available on request from the corresponding author.
